# Procalcitonin as a marker of bacterial sepsis in immunocompromised patients

**DOI:** 10.1186/1742-4690-9-S1-P90

**Published:** 2012-05-25

**Authors:** Lana Gatserelia, Lali Sharvadze, Marine Karchava, Nino Babridze, Tengiz Tsetvadze, Natia Dvali, Lela Dzigua, Nika Chxartishvili

**Affiliations:** 1 Head of Virology Lab. at Infectious Diseases, Aids and Clinical Immunology Research Center, Tbilisi, Georgia

## Introduction

Procalcitonin (PCT) is a recently described marker of severe sepsis. It was decided to assess the value of PCT as a marker of secondary infection in patients infected with HIV in Georgian AIDS Center.

## Materials and methods

PCT plasma levels were measured by quantitative assay BRAHMS-Biomérieux using the VIDAS analyser in a prospective study in 135 HIV-infected individuals: 87 asymptomatic and 48 with lever or suspected secondary infections.

## Results

The baseline plasma level of PCT was (0.5 ng/ml +/- 0.5), even in the latest stages of the disease, and did not differ from the values of healthy subjects (0.54 ng/ml +/- 0.1). EDTA-treated whole blood was collected from patients before starting specific antimicrobial therapy. No elevation of PCT level was detected in HIV-infected patients with evolving secondary infections including PCP (n = 4), cerebral toxoplasmosis (n = 5), viral infections (n = 9), mycobacterial infections (n = 4), localized bacterial (n = 13) and fungal infections (n = 4), and in various associated infectious and non-infectious febrile events (n = 15). All these plasma values were lower than 2 ng/ml. In contrast, high PCT plasma levels were detected in one HIV-infected patient with a septicaemic influenza infection (17 ng/ml) and another one with a septicaemic Pneudomonas aeruginosa infection (46 ng/ ml), PCT values decreased rapidly under appropriate therapy.

## Conclusions

We found that PCT is a specific marker of bacterial sepsis in HIV-infected patients, as no increase in other secondary infections could be detected in those patients. A rapid determination of PCT level could be useful to verify or refute bacterial sepsis for a better management of febrile HIV-infected patients.

